# Editorial: Preclinical studies exploring the central and peripheral mechanisms of cancer pain

**DOI:** 10.3389/fpain.2022.1121765

**Published:** 2023-01-09

**Authors:** Marta Diaz-delCastillo, George Latimer Wilcox

**Affiliations:** ^1^ Department of Forensic Medicine, University of Århus, Århus, Denmark; ^2^ Department of Drug Design and Pharmacology, University of Copenhagen, Copenhagen, Denmark; ^3^ Departments of Neuroscience, Pharmacology and Dermatology, University of Minnesota Twin Cities, Minneapolis MN United States

**Keywords:** cancer pain, translational, animal models, neuronal sprouting, bone cancer pain (BCP), bone cancer pain model, HNSCC (head and neck squamous cell carcinoma), oral cancer (OC)

**Editorial on the Research Topic** Preclinical studies exploring the central and peripheral mechanisms of cancer pain

## Introduction

It is estimated that 19.3 million new cancer cases were diagnosed worldwide in 2020 (1), and up to 28.4 million new cancer diagnoses are predicted for 2040 (1). Despite growing advances in disease treatment, pain remains among the most common and feared cancer symptoms, affecting 30% to 66% of patients depending on the disease stage (2). These estimates are expected to exponentially increase following the continuous advances in cancer treatment that promote longer survivorship.

In comparison, developments in analgesic therapies for the treatment of cancer pain have been few and far between. The challenges in the study and treatment of cancer pain are many, due to its heterogeneity in localization (oral, bone, visceral), cause (disease-induced vs. treatment-induced), magnitude, and responsiveness to analgesics, on top of the individual psychological burden that it poses for each patient. To better understand the mechanisms of cancer pain, a variety of animal models have been developed since 1998, when Schwei et al. implanted fibrosarcoma cells in the intramedullary cavity of a mouse femur (3) and Wacnik et al. implanted these cells into mouse calcaneus bone (4). This revolutionary approach translated into reproducible models where behavioural tests allowed the detection of a nociceptive phenotypes; this had been previously hard to achieve, as available cancer models depended on intracardiac cancer cell administration, which led to heterogeneity in metastatic sites and size. In their narrative review, Haroun et al. provide a comprehensive overview of the variety of animal models that have since been developed to study cancer pain. The review digs into murine models of cancer-induced bone pain, chemotherapy-induced neuropathic pain, as well as models of non-bone cancers, including pancreatic and oral squamous cell carcinoma. Moreover, Haroun et al. discuss some of the current and upcoming cancer pain analgesics, summarizing some of the emerging targets that may pose novel treatment avenues in the near future.

The wide range of available animal models of cancer pain reflects the need for different modelling systems that allow the study of the diverse contributing nociceptive factors. As an example, murine models of head and neck squamous cell carcinoma largely rely on xenograft transplants (5); these models are highly valuable, as they allow maintenance of inherent genomic alterations in the human cancer cells, but fail to reproduce the role of the immune system in the host microenvironment. To circumvent this limitation, Horan et al. evaluated the differential nociceptive phenotype induced by tongue inoculation of two oral cancer cell lines with different immunogenicity potential. Their characterization of pain-like behaviours and neuronal plasticity revealed two very distinct phenotypes dictated solely by tumour immunogenicity, highlighting the important and complex role of the host immune system in cancer pain.

Another major contributor to cancer pain arises from chemotherapeutic treatments that induce nerve damage. Chemotherapy-induced neuropathic pain is highly prevalent, affecting up to 68% of cancer patients in the first month after treatment, and approximately 30% after 6 months (6). Due to the high prevalence of this occurrence, there is an unmet need to identify the specific mechanisms that may translate into better agents with fewer side effects. Caudle and Neubert evaluated the effect of oxiplatin and paclixatel (common chemotherapeutics in the treatment of colorectal and breast cancer, respectively) on TRPM8-expressing trigeminal ganglion neurons. The specific agents were selected due to their differential toxicity, as oxiplatin is well known to induce cool face sensitivity, whereas paclixatel does not. Using whole cell voltage clamp, their study demonstrated that both chemotherapeutic agents increase hyperpolarization-activated cyclic nucleotide-gated channels and voltage-gated sodium channels, but only oxiplatin induced peripheral damage to the TRPM8-expressing axons. The authors interpret that oxiplatin-induced peripheral nerve degeneration is ultimately responsible for the facial cool sensitivity derived from this drug. Understanding the mechanisms of chemotherapy-induced neuropathic pain will help shape novel analgesic agents of combination therapies that lessen the burden of anti-cancer treatment on quality of life.

While animal studies can provide crucial information on the underlying mechanisms of cancer pain, proof-of concept studies in human tissue are unavoidable to validate the findings. Hansen et al. provide critical evidence on the microenvironmental alterations induced by cancer metastasis to the bone using biopsies from breast cancer patients. Indeed, breast cancer is characterized by its organotrophic metastasis to bone, which leads to the development of cancer-induced bone pain in over 80% of patients at diagnosis (7). Already in 1889, Steven Paget proposed the “seed and soil” theory (8), where the cancer cells (“seed”) metastasize to appropriate microenvironments (“soil”), which become tumour-permissive and undergo cellular and molecular modifications that support cancer growth (9). Further modifications to the microenvironment entail following metastatic bone invasion and proliferation of cancer cells, including tumour-induced alterations to bone innervation, which have been correlated to nociception in animal models of cancer-induced bone pain (10, 11, 12, 13). Hansen et al. provide the first evidence of cancer-induced alternations to bone innervation in human, by analysing the density of nerve profiles present in bone biopsies from healthy and breast cancer female patients at different disease stages. As in animal models (12), cancer infiltration induced nerve sprouting in the bone marrow of breast cancer patients with bone metastasis, and the nerve profiles of these patients were not located as close to vascular structures as those of healthy controls. Given the animal evidence that suggests blockade of nerve sprouting as an analgesic target for cancer-induced bone pain (11, 12), this study provides crucial proof-of-concept data to support this research direction.

Overall, the articles contained within this Research Topic highlight the burden of cancer pain and compile a variety of animal and proof-of-concept human studies that will help tackle this growing health concern. Increasing our mechanistic understanding of cancer-induced bone pain remains an important step in the development of novel analgesics so that cancer survivors cannot only live longer, but also enjoy their quality of life.
